# The Effect of Pre-Condition Cerebella Fastigial Nucleus Electrical Stimulation within and beyond the Time Window of Thrombolytic on Ischemic Stroke in the Rats

**DOI:** 10.1371/journal.pone.0128447

**Published:** 2015-05-27

**Authors:** Weiju Tang, Weiwei Dong, Peng Xie, Pengfei Cheng, Shunjie Bai, Yifei Ren, Gong Wang, Xiuying Chen, Chun Cui, Yuxiang Zhuang, Wen Huang

**Affiliations:** 1 Department of Neurology, Xinqiao Hospital, Third Military Medical University, Chongqing, China; 2 Department of Neurology, The First Affiliated Hospital of Chongqing Medical University, Chongqing, China; 3 Department of Radiology, Xinqiao Hospital, Third Military Medical University, Chongqing, China; University of Kansas, UNITED STATES

## Abstract

**Objective:**

To investigate the effect of neurogenic neuroprotection conferred by cerebellar fastigial nucleus stimulation (FNS) and the role of PPARγ- mediated inflammation in a rat model of cerebral ischemia reperfusion.

**Methods:**

After a continuous 1 hour fastigial nucleus electric stimulation, the male Sprague Dawley (SD) rats were given middle cerebral artery occlusion (MCAO) for 1, 3, 6, 9, 12 and 15 hours undergoing reperfusion with intravenous recombinant tissue plasminogen activator (rt-PA), while the control group received without FNS. After 72h of reperfusion, the neurological deficits, infarct volume and brain edema were evaluated. The brain tissue in ischemic penumbra was determined the myeloperoxidase (MPO) activity by a spectrophotometer and expression of PPARγ was measured by Rt-PCR and Western blotting.

**Results:**

Our findings showed that FNS group had significantly reduced infarct volume and brain edema, and improved neurological deficits compared with the control group, especially in 6h and 9h reperfusion subgroups(p<0.05). The expression levels of PPARγ increased gradually and the peak may be before and after 9h reperfusion, the 3h, 6h, 9h, 12h and 15h reperfusion subgroups were higher than each control group(p<0.05). The MPO activity of 6h, 12h and 15h reperfusion subgroups were higher than each control group(p<0.05).

**Conclusions:**

The neuroprotective effects of FNS have been shown to prolong the therapeutic window in cerebral ischemia/reperfusion, which might be related to the PPARγ mediated-inflammation in penumbral region.

## Introduction

Ischemic cerebrovascular disease is highly prevalent worldwide and one of the leading causes of death and permanent disability and has increased the financial burden on patients and society [1]. The increasing incidence and younger of ischemic stroke is related to harmful habits, the ageing of society. Prevention and treatment of ischemic stroke face more seriously situation. Intravenous recombinant tissue plasminogen activator (rt-PA) is the only approved treatment for acute cerebral ischemic within 4.5 h from symptoms onset, as currently approved treatments by Food and Drugs Administration (FDA) and supported by observational studies [2]. However, many patients are still left undertreated due to the main fact of the narrow time window. Beyond the time window of thrombolytic therapy, it will do more harm with an exacerbation of cerebral tissue injury and a profound inflammatory response [3]. In other words, cerebral ischemia/reperfusion (I/R) can cause neurovascular injury, leading to cerebral edema and brain hemorrhage. Various mechanisms are involved in cerebral ischemia/reperfusion (I/R), such as inflammation and apoptosis [4]. The functional recovery of lesion area required oxygen and other nutrients by cerebral blood flow recanalization, therefore, revascularization is necessary [5]. It is also mean that extending the time window of thrombolytic is also essential. Thus, effective prevention and controlling of cerebral ischemia/reperfusion (I/R) injury is a pressing concern. These findings [6–7] suggest electrical stimulation of the cerebellar fastigial nucleus in rat confers remarkable protection from brain injury. Thus, FNS attenuates the brain damage produced by cerebral ischemia and possesses the intrinsic capability of self-protection against injury. However, little is known about the effects of pre-FNS on time window of thrombolytic therapy. Therefore, we sought to determine whether FNS exerts its neuroprotective effect to prolong the therapeutic window in cerebral ischemia/reperfusion and related mechanisms.

## Materials and Methods

### Ethics Statement

The rats were housed and maintained at room temperature (22±2°C) and humidity (<40%) with free access to food and water. All animals care and experimentation were approved by the Institutional Animal Care and Use Committee of the Third Military Medical University (license No. SYXK (Yu) 2012–0011). Efforts were made to reduce the number of animals used and minimize animal suffering. All surgery was performed under chloral hydrate anesthesia. All mice were decapitated by deep of anesthesia.

### Experiment animals

Male Sprague—Dawley rats, of clean grade, weighing 270±30 g, were provided by the Experimental Animal Center of Third Military Medical University, Chongqing, China. The rats were divided FNS group with pre-FN stimulation for 1 h before the cerebral I/R and control group with only cerebral I/R by random number method. All the rats were given middle cerebral artery occlusion for 1, 3, 6, 9, 12 and 15h undergoing reperfusion with rt-PA.

### Cerebellar fastigial nucleus stimulation

Electrical stimulation of the fastigial nucleus was performed according to a previously described method [8], but with slight modifications. Rats were fasted for 8–12 hours and then anesthetized with intraperitoneal injection of 3.5% (w/v) chloral hydrate (10 mL/kg). After a calvarium thoracic incision was made, the fastigial nucleus was accurately positioned using a stereotaxic atlas of the rat brain. With the posterior border of the anterior fontanelle set as the zero point, an 11.6 mm incision was made, with a 1.2 mm incision on the left side of the median line. A hole (5.6 mm deep) was made in the skull for the attachment of electrodes. A 50 μA direct-current square-wave pulse (0.5 ms; 70 Hz) was applied. We thought electrical stimulation successed by observing phenomenen of increased blood pressure, wagging tail and piloerection. Once the electrode was securely in place at the FN, the stimulation was started. Each electrical stimulation lasted 1 hour during light anesthesia.

### Middle Cerebral Artery Occlusion and Reperfusion (MCAO/R)

The method was performed according to Busch [9] previously described.

Thromboembolic occlusion of the left middle cerebral artery (MCA) was achieved by injection of 12 autologous blood clots into the internal carotid artery (ICA). At the beginning of the experiment, 0.6 ml fresh venous blood were mixed with 75u thrombin and immediately injected into 50 cm pieces of PE 50 catheter inner diameter 0.53mm. These segments were cut into twelve small pieces, producing cylindrical clots of about 2.5 mm size in phosphate-buffered saline. After full clot retraction was completed at 4 h, the clots, together with the albumin solution, were drawn up into a metre long PE 50 catheter. After surgical preparation of the left carotid artery and ligation of the pterygo-palatine artery, a PE 50 catheter was inserted into the external carotid artery (ECA). The tip of the catheter was placed close to the carotid bifurcation. The 12 clots were injected one after another over a period of 30 s, during which the common carotid artery (CCA) was temporarily closed. The rats were given rt-PA (5 000 U/kg) at each time for reperfusion. During the whole surgical procedure, the room temperature was monitored and maintained at 37–38°C with a heating pad. After surgery rats put return to cages, free diet and penicillin water.

### Neurological deficit evaluation

After 72 h of reperfusion, neurological deficit was scored according to the 5-point scoring system described by Zea Longa [10]. The scoring system was used as follows: (1) a score of 0: normal walk or no neurologic deficit; (2) a score of 1: failure to extend opposite forepawfully or a mild focal neurologic deficit; (3) a score of 2: circling to the contralateral side or amoderate focal neurologic deficit; (4) a score of 3: falling to the contralateral side or a severe focal neurologic deficit; and (5) a score of 4: no spontaneous walking with depressed consciousness level. The neurological evaluation was performed by an investigator who was blinded to the study protocol. Rats with a score of “0” or “4” were excluded. Successful ischemia/reperfusion was confirmed by triphenyltetrazolium chloride staining, which indicated the infarct focus.

### Determination of MPO activity

After neurological deficit evaluation (n = 5 at each time point), the animals were anesthetized with chloral hydrate and brains and decapitated. Brain samples were taken from the ischemic hemisphere of the left middle cerebral artery (MCA) area and stored at −80°C for later analysis. The spectrophotometer method was applied to determine myeloperoxidase (MPO) activity [11]. MPO levels in the ischemic hemisphere tissues were measured with a rat MPO assay kit (Jiancheng Bioengineering Institute, Nanjing, China) using a spectrophotometer at 460 nm for 2 min. One unit of MPO activity was defined as the amount of enzyme degrading 1 mmol of peroxidase/min at 25°C. MPO activities in the ischemic hemisphere tissues were calculated by using a standard curve.

### Western blot analysis

Western blot was performed according to previously described methods [12]. The ischemic hemisphere tissues were prepared in an ice-cold lysis buffer. The samples were lysed in protein lysis buffer. Protein concentration was measured using a colorimetric protein assay kit. Briefly, proteins of 20 μg were separated on SDS-polyacrylamide gels (SDS-PAGE) and transferred onto a nitrocellulose membrane. The membranes were incubated overnight at 4°C with PPARγ-anti-rabbit IgG (1:500). Subsequently, the membranes were incubated for 1 hour with secondary antibodies, and band detection was performed using the enhanced chemiluminescence detection kit. The densitometric analyses were quantified using an Image Lab Software (Bio-Rad) analysis system.

### Reverse transcription polymerase chain reaction (Rt-PCR)

The PPARγ sense strand: Forward Primer: 5'TGCGGAAGCCCTTTGGTGAC 3', Reverse Primer: 5'GCAGCAGGTTGTCTTGGATGTC 3'. Primer sequences were synthesized from Chongqing Haiyun Company. The details of RNA extraction refer to "RNA extraction and purification" section [13] form the "Molecular Cloning". Sample volume for RNA extraction was 20μl, using 3μl template, 10×2μl buffer, 4μl MgCl_2_ (25mmol/L), 2μl dNTP (10mmol/L), 0.5μl Enzyme inhibitor (40U/μl), 1μl AWV Reverse transcriptase (5 U/μl), 1μl Oligo dT-Adaptor primer (2.5pmol/μl), and 6.5μl enzyme water. It was stored at 4°C for future use, add the template and water of degeneration at 72°C, and then add the rest to reaction, 72°C for 2.5 min, 42°C for 40 min, 95°C for 5 min. Fluorescence quantitative PCR sample volume and reaction conditions: sample volume: 1μl template, 10×5μl buffer, 5μl MgCl_2_ (25mmol/L), 1μl dNTP(10mmol/L), sense chain: 0.8μl (20pmol/μl), 0.8μl antisense strand (20pmol/μl), 0.4μl probe (20pmol/μl), 0.5μl taq polymerase (5 U/μl), adding H_2_O to 50 μ l of reaction system. The efficiency of the PCR reaction was evaluated for each amplified conditions. The reaction conditions were as follows: 94°C for 2 min, 94°C for 10 sec, 53°C for 30 sec, 72°C for 40 sec, repeating 45 cycles, 72°C for 10 min.

### Measurement of infarct volume

The 2,3,5-triphenyltetrazolium chloride (TTC) staining was used to check the brain infarction size [14]. The rats were anesthetized and sacrificed after the neurological examination after 72 h of surgery (n = 5 at each time point). Then the brains were carefully removed from the skull and kept at −20°C for 10min. Frozen brains were sliced into consecutive 2 mm coronal sections and immersed in a 2% TTC solution for 30 min at 37°C. The infarct areas were photographed using a digital camera with a high resolution. Image analysis software was applied to the measurement of the infracted area.

### Brain edema

Brain water content was measured by the standard wet—dry method. After 72 h of reperfusion, rats were decapitated and brains were immediately removed from the skull. Before TTC staining, the two hemispheres were weighed (wet weight), separately. Then the tissues were dried at 105°C for 24 h to determine the dry weight after staining. The degree of brain edema was calculated by the following equation: water content = (wet weight− dry weight) / wet weight × 100%.

### Statistics

Data were expressed as mean ± standard deviation. The Statistical Package for Social Sciences (SPSS) 19.0 software was used for statistical analyses by Student's t-test. A probability (p)-value < 0.05 was considered statistically significant.

## Results

### FNS improved neurological deficit

**Fig 1:**
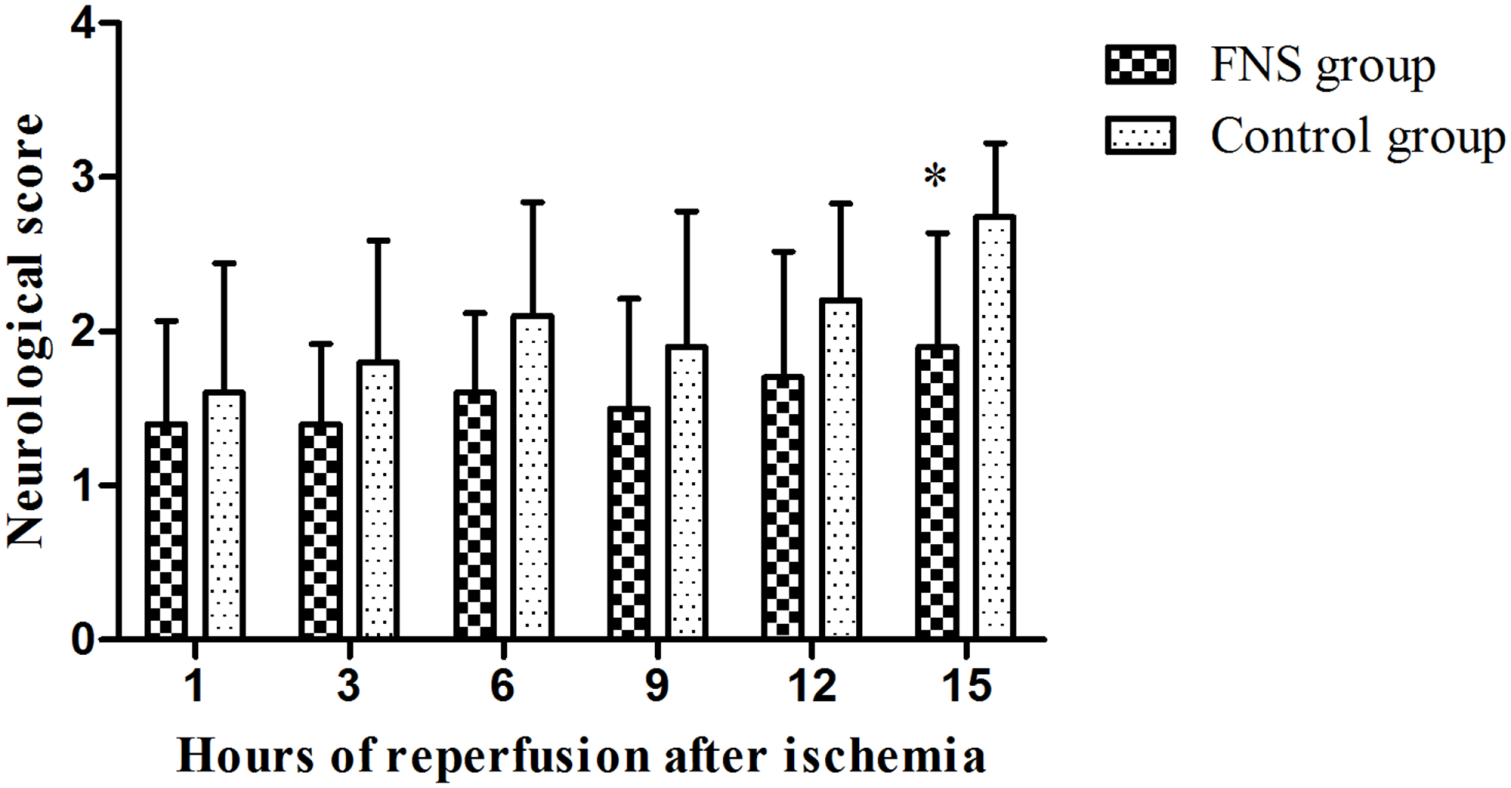
The scores of two groups. FNS group were significantly decreased compared to the control group. Results are expressed as the mean ± SD. *p < 0.05, vs the control group, n = 10.

After 72h of reperfusion, the neurologic deficit scores were evaluated and presented in Fig 1 and S1 File. As results shown, neurologic deficits increased gradually in the two groups. In 15h reperfusion subgroup, the scores of FNS group (1.90±0.54) were significantly decreased compared to the control group (2.74±0.55). (*p*<0.05).

#### The mortality rate

**Fig 2:**
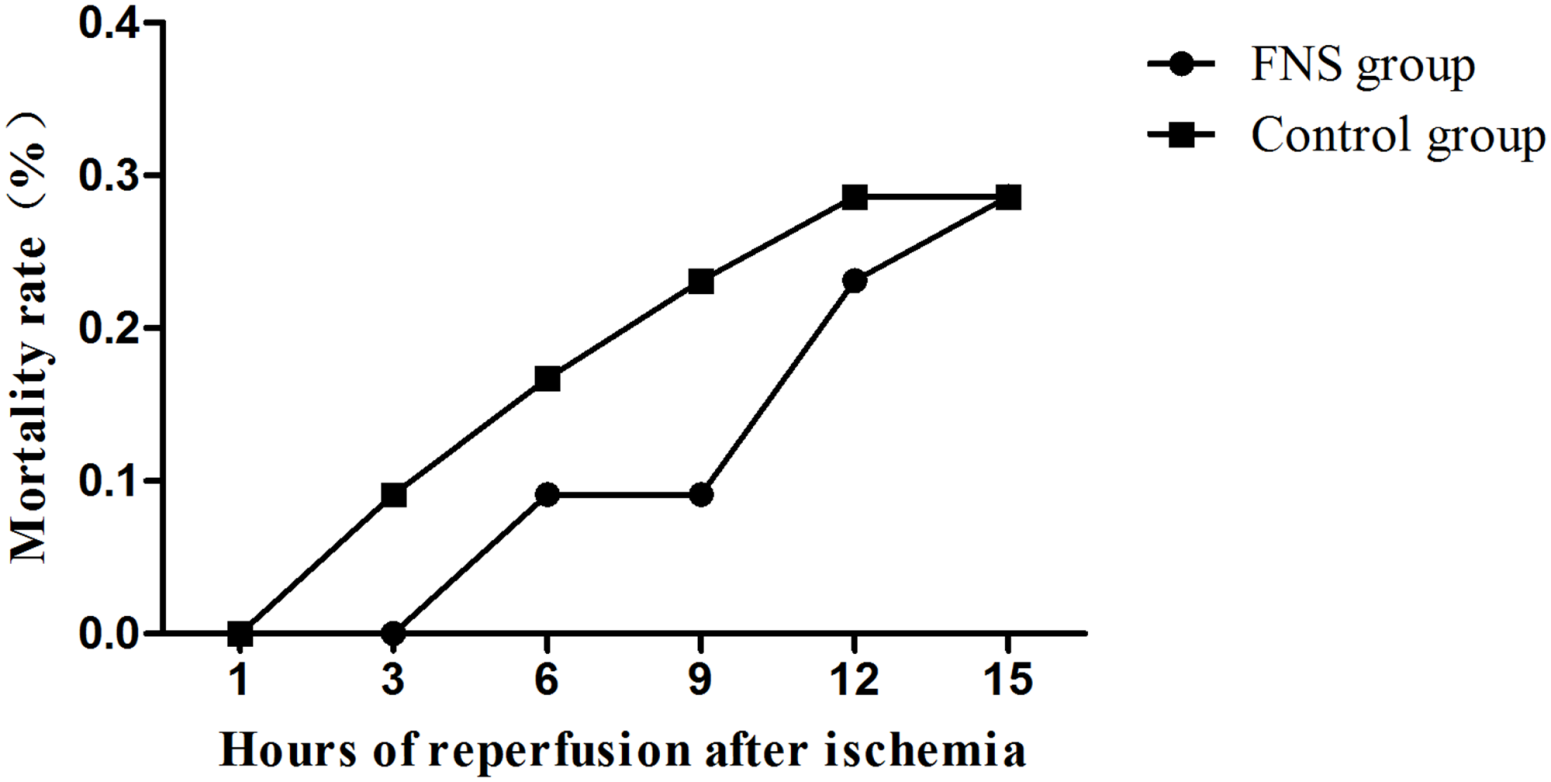
The mortality of two groups. Every group has 10 animals to be survival.

After 72h of reperfusion, the decreased mortality rate in FNS group presented in Fig 2. In 3h reperfusion subgroup, 1 animal had died in the control group and no animals died in FNS group. In 6h reperfusion subgroup, 2 animals had died in the control group and 1 animal died in FNS group. In 9h reperfusion subgroup, 3 animals had died in the control group and 1 animal died in FNS group. In 12h reperfusion subgroup, 4 animals had died in the control group and 3 animals died in FNS group.

### FNS up-regulated the expression of PPARγ

**Fig 3:**
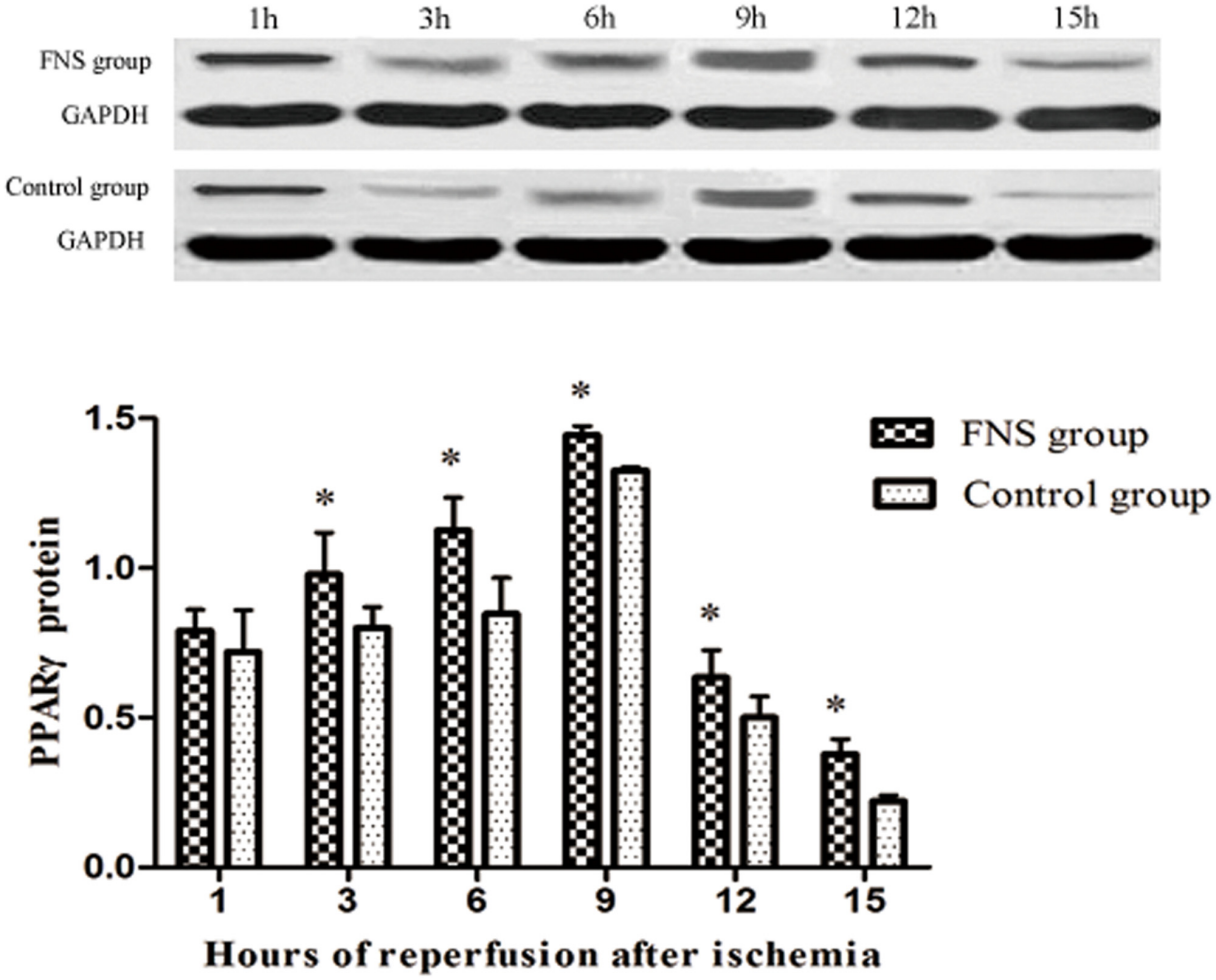
The protein expressions of PPARγ in cerebral ischemic penumbra. Results are expressed as the mean ± SD. *p < 0.05, vs the control group, n = 5.

**Fig 4:**
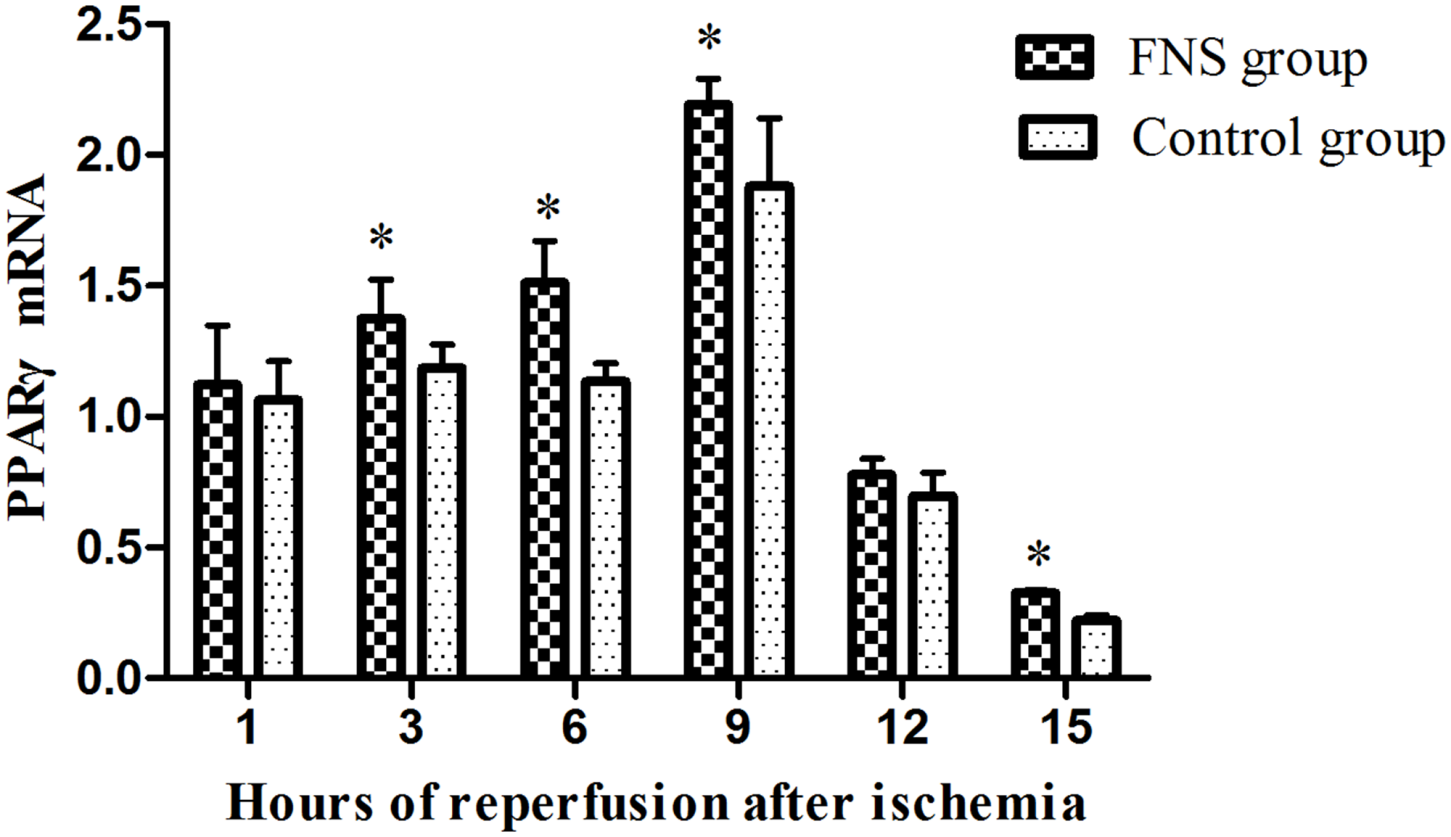
The mRNA levels of PPARγ in cerebral ischemic penumbra. Results are expressed as the mean ± SD. *p < 0.05, vs the control group, n = 5.

To further analyze FNS-induced anti-inflammatory effects, we also examined the expression of PPARγ using Western-blot (S2 File) and Rt-PCR. The results suggested that PPARγ levels were mainly located in the 6h and 9h reperfusion subgroup (Figs 3 and 4). Compared with control group, the levels of PPARγ was significantly higher in 3h, 6h, 9h, 12h and 15h reperfusion subgroup (*P* < 0.05). Rt-PCR analysis further confirmed this result. A significantly higher expression of PPARγ were found in the FNS group compared with the control group at 3h、6h、9h、15h reperfusion.

### FNS inhibited inflammatory reaction in cerebral ischemia/reperfusion

**Fig 5:**
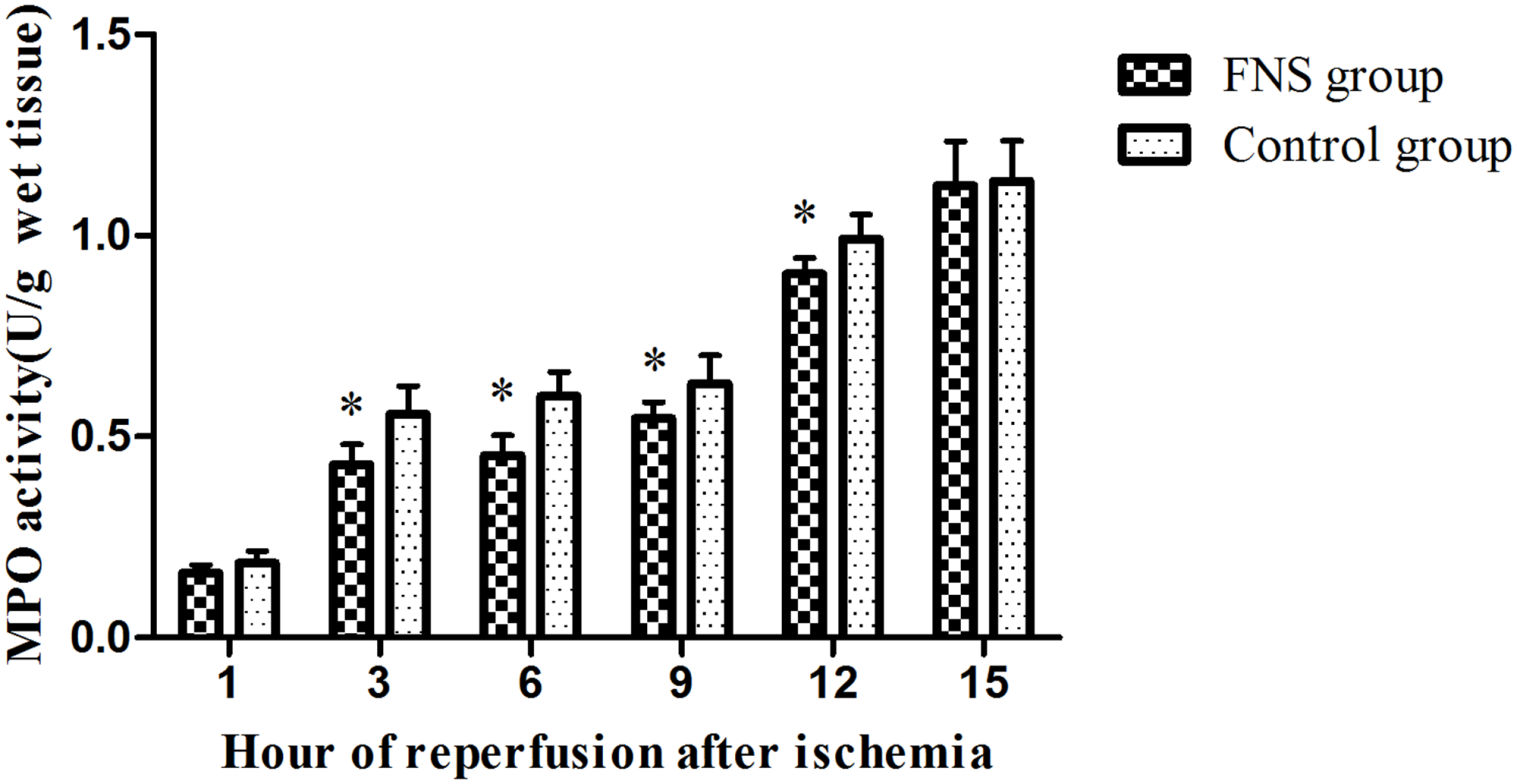
The MPO activity was detected using a spectrophotometer in cerebral ischemic penumbra. Results are expressed as the mean ± SD. *p < 0.05, vs the control group, n = 5.

To investigate the effects of FNS on inflammatory reaction in cerebral ischemia/reperfusion, we determined the MPO activity by spectrophotometer method. As depicted in Fig 5, the MPO activity was significantly higher in the control group than those of the FNS group at 3h, 6h, 9h and 12h reperfusion (*P* < 0.05). Compared with control group, FNS has shown obvious signs of alleviating the increase of inflammatory mediators in a time-dependent manner.

### FNS decreased ischemic infarcted area and brain edema

**Fig 6:**
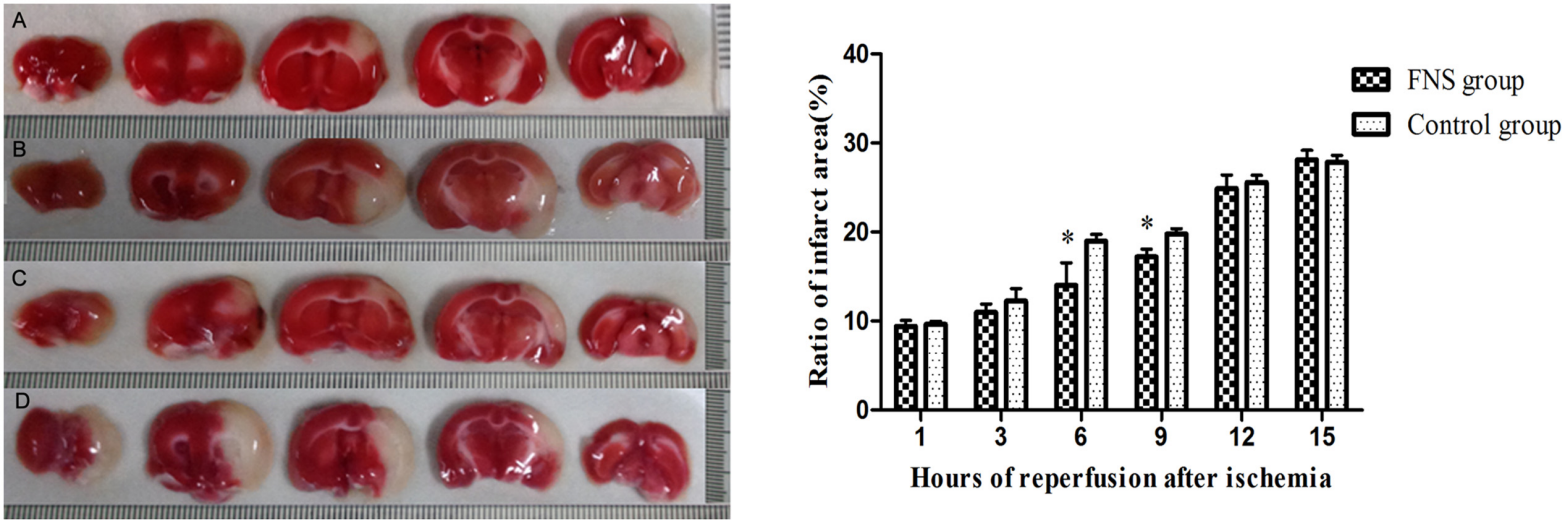
The measurement of infarct volume by TTC staining. (A): the 6h FNS group; (B): the 6h control group; (C): the 9h FNS group; (D): the 9h control group. The infarct area was shown as white color, and FNS significantly reduced the infarct volume. Results are expressed as the mean ± SD. *p < 0.05, vs the control group, n = 5.

**Fig 7:**
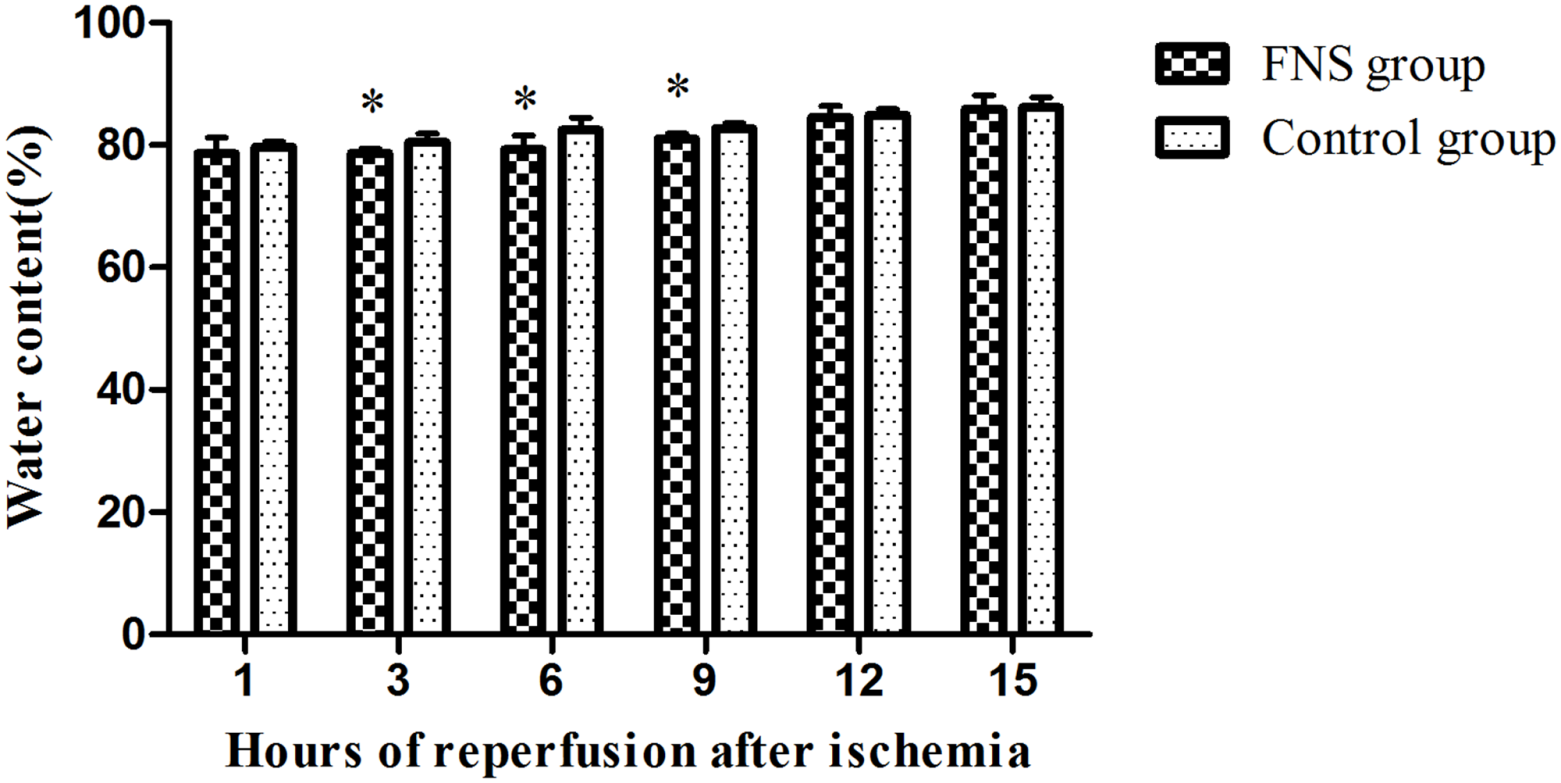
Brain water content of two groups. (A) The percentage of ischemic lesion area was represented as the ratio of the infarction area to the whole slice area. (B)Water content was calculated by (wet weight −dry weight) /wet weight × 100%. Results are expressed as the mean ± SD. *p < 0.05, vs the control group, n = 5.

Infarct tissues were visualized as an area of unstained part in the ischemia/reperfusion group, in contrast to the viable tissue, which stained red. As shown in Figs 6 and 7 and S3 File, in the FNS group, the infracted volume and the brain water content were significantly reduced as compared with the control group, especially at 6h and 9h reperfusion (*P* < 0.05).

## Conclusions

Ischemic cerebrovascular disease is as the third most common cause of death in the developed countries [15]. Stroke is a terrifying ordeal that always despoils people of independent living by damaging language, movement and cognition without warning [16]. To reduce tissue damage from acute cerebral ischemia and to improve the neurological status of patients, the use of rt-PA is the main measure within the time window [17]. However, many patients are still short of treatment, mainly due to the narrow time window and other contraindications for rt-PA. Beyond the time window of thrombolysic therapy significantly increased the risk of cerebral hemorrhage and severe brain edema caused by reperfusion-induced brain damage, and this risk increases with progressive delays in treatment initiation seriously. The cerebral blood flow recanalization is significant for patients. Therefore, seeking effective measures to extending the time window of thrombolysis focal cerebral ischemia has attracted more attention. Many researchers [18–20] suggested that FNS can induce neuroprotective effect against cerebral ischemia. FNS has been finished product and convenient use, no adverse reaction and many other advantages. We focus on those mechanisms and the clinical applications of FNS in extending the time window of thrombolysis.

A cerebral middle cerebral artery ischemia/reperfusion model [5] by self-thrombus was used in the study. This study observed that FNS could improve neurological deficit, decreased ischemic infarcted area and brain edema. FNS reduced damage that might account for the neuroprotection. The three observed indicators were significantly reduced as compared with the control group, especially at 6h and 9h reperfusion subgroup(*p*<0.05). The data indicated that pre-stimulation may be able to improve the prognosis of thrombolysis beyond the time window. Recently many investigations [21–22] supported the idea that cerebral ischemia triggered the inflammatory response which may further amplify tissue damage. Inflammatory response on the damage of cerebral tissues after ischemia had become a focus, playing a crucial role in the necrosis and apoptosis of nerve cells and resulting in secondary brain damage [23]. After cerebral ischemia/reperfusion (I/R), inflammatory response is involved in secondary brain injury including the rapid activation of resident microglial cells and infiltration of neutrophils and macrophages into the injured area.

Later researches [24–25] suggested that PPARγ exhibits antiinflammatory properties by regulating negatively the expression of various pro-inflammatory molecules including IL-6 and tumor-necrosis factor-a (TNF-a). Evidence accumulated also shows the level of PPARγ in the glial cells and central nervous system [26]. The tissues of ischemic hemisphere were determined according to the prior literature [27]. The mRNA and protein levels of PPARγ in the tissue of ischemic hemisphere are regarded as that of tissue of penumbra. Cerebral ischemia results in an irreversibly damaged ischemic core and rescued surrounding penumbra tissue. The cerebral ischemia penumbra tissues were made to detect the expression of PPARγ. The results suggested that PPARγ levels were significantly higher in the 6h and 9h reperfusion subgroup, compared with control group, the expression levels of PPARγ increased gradually and the peak may be before and after 9h reperfusion and then reduced. This indicated that FNS up-regulated the expression of PPARγ to protect the rats avoid the cerebral ischemia injury. PPARγ agonists have shown promising beneficial effects in several animal models of central nervous system disorders in which an inflammatory component is strongly implicated [28–29]. On the other hand, it aiso indicated that PPARγ antiinflammatory effect in penumbral region plays an important role in brain ischemia. Another studies [30–31] also proved that PPARγ mediated neuroprotection after cerebral infarction.

MPO is a marker enzyme for the inflammatory cells, majorly neutrophil derived. MPO activity reflects inflammatory cells infiltration, especially neutrophil, and correlates well with movement into brain tissues [32]. By measuring its activity, accumulation and infiltration of neutrophils after cerebral ischemia can be estimated. In our results, the MPO activity gradually increased in the two groups by a time-dependent manner, but the FNS group was lower than that in control group beyond the time window of rt-PA. It suggested that FNS still has certain protective effect but not significant beyond the time window of rt-PA, this may also suggest that antiinflammatory reaction mediated by FNS in cerebral ischemic penumbra may have a delayed effect.

The results of this study should be considered its limitations. First, successful ischemia/reperfusion was confirmed by triphenyltetrazolium chloride staining, which indicated the infarct focus, not the cerebral blood flow. Another limitation is that the sample size was small which may have affected the power to detect a statistically significant difference in short-term outcomes in both groups. A third is that other mechanisms about cerebral ischemia-reperfusion have not involved in this study. To fulfill an increasing need for an effective and practical intervention strategy, it is important to understand the mechanisms underlying a potent neuroprotection.

In conclusion, our study indicate that pre-FNS produces neuroprotective effects via inhibiting inflammatory response in cerebral ischemia/reperfusion rats beyond the time window of thrombolysis, but not significant. The mechanisms underlying the protection exhibited by FNS seem to involve the up-regulated the expression of PPARγ and inhibition for neutrophil infiltration to against inflammatory damage. FNS would be desirable to improve the efficacy and safety of stroke therapy and to prolong the effective time window of it after stroke. Thereby, more patients become eligible for receiving this treatment. However, additional clinical researches and further clarify the effect of FNS are needed to determine the effect.

## Supporting Information

S1 File The original data of neurological score. (DOC)

S2 File The original data of neurological score. This file provides pictures of gels and the sequence about WB.
(DOC)

S3 File The original pictures of TTC staining. (RAR)
