# Supplementary material for: The Effect of Pre-Condition Cerebella Fastigial Nucleus Electrical Stimulation within and beyond the Time Window of Thrombolytic on Ischemic Stroke in the Rats
Source: PLoS One. 2015 May 27;10(5):e0128447. doi: 10.1371/journal.pone.0128447 (PMC4446308; doi:10.1371/journal.pone.0128447)
Supplement: S1 File — (DOC) [file pone.0128447.s001.doc]

1h reperfusion subgroup: FNS group: 1-1-2-1-1-1-3-2-1-1-2

Control group: 1-1-2-2-1-1-1-3-3-1

3h reperfusion subgroup: FNS group: 2-2-1-1-2-2-1-1-1-1

Control group: 3-3-2-2-1-1-2-2-1-1

6h reperfusion subgroup: FNS group: 2-2-2-1-2-1-1-2-2-1

Control group: 3-3-1-3-2-1-1-2-2-2

9h reperfusion subgroup: FNS group: 3-1-2-2-1-1-2-1-1-1

Control group: 1-3-3-2-2-2-1-3-1-1

12h reperfusion subgroup: FNS group: 1-1-2-2-1-1-2-3-3-1

Control group:1-2-2-3-3-3-2-2-2-2

15h reperfusion subgroup: FNS group: 2-1-2-3-2-3-2-2-1-1

Control group:3-2-2-2-3-3-3-3-3-3
