# Supplementary material for: The Effect of Pre-Condition Cerebella Fastigial Nucleus Electrical Stimulation within and beyond the Time Window of Thrombolytic on Ischemic Stroke in the Rats
Source: PLoS One. 2015 May 27;10(5):e0128447. doi: 10.1371/journal.pone.0128447 (PMC4446308; doi:10.1371/journal.pone.0128447)
Supplement: S2 File — This file provides pictures of gels and the sequence about WB. (DOC) [file pone.0128447.s002.doc]

1.


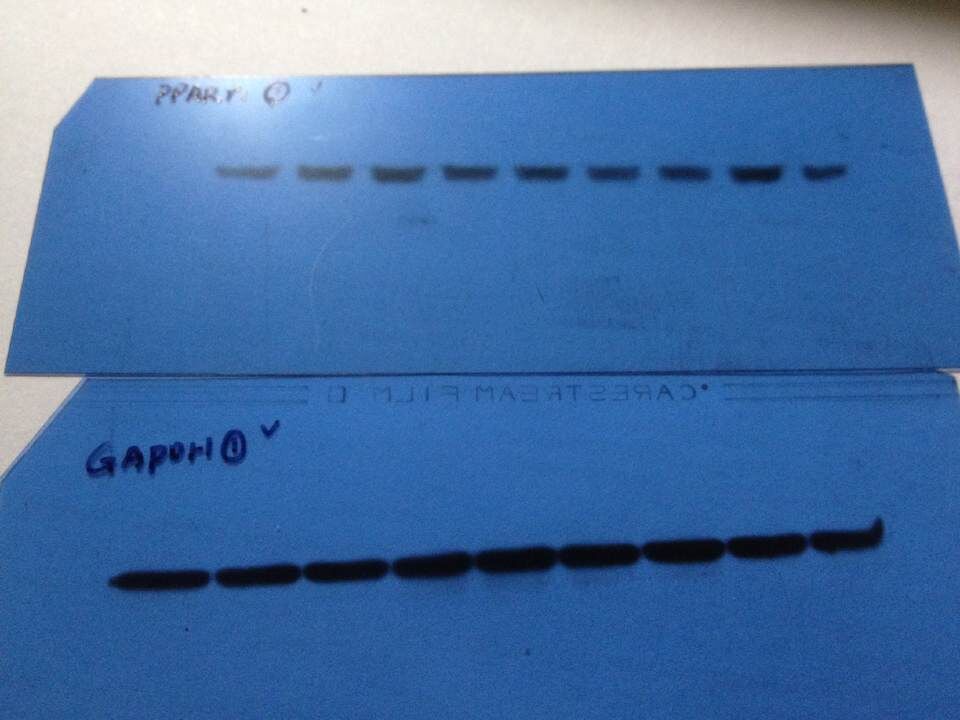


2.


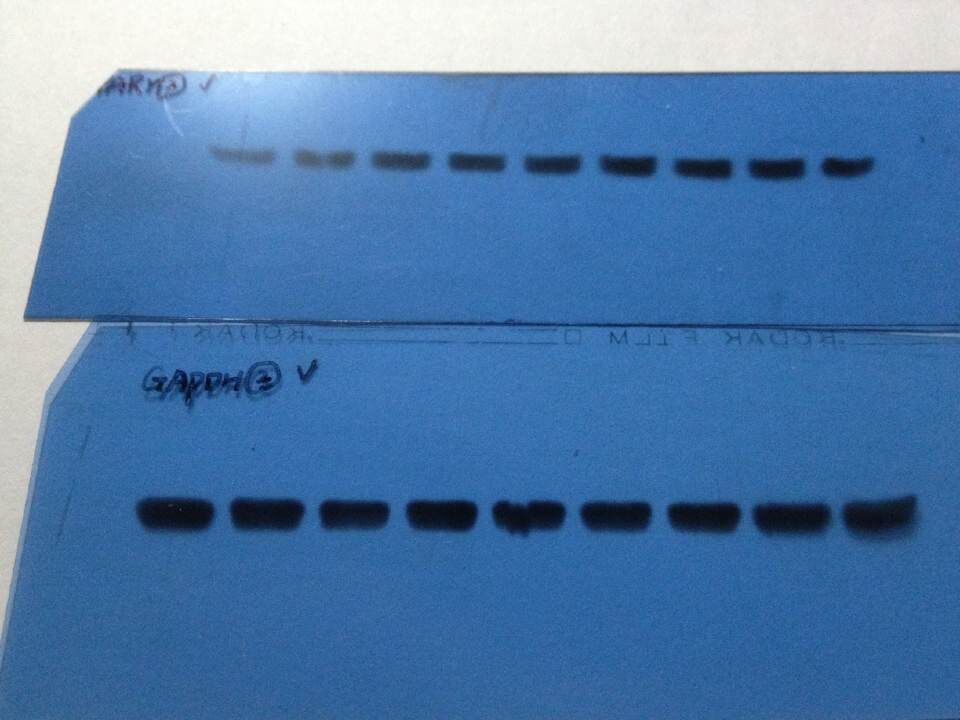


3.


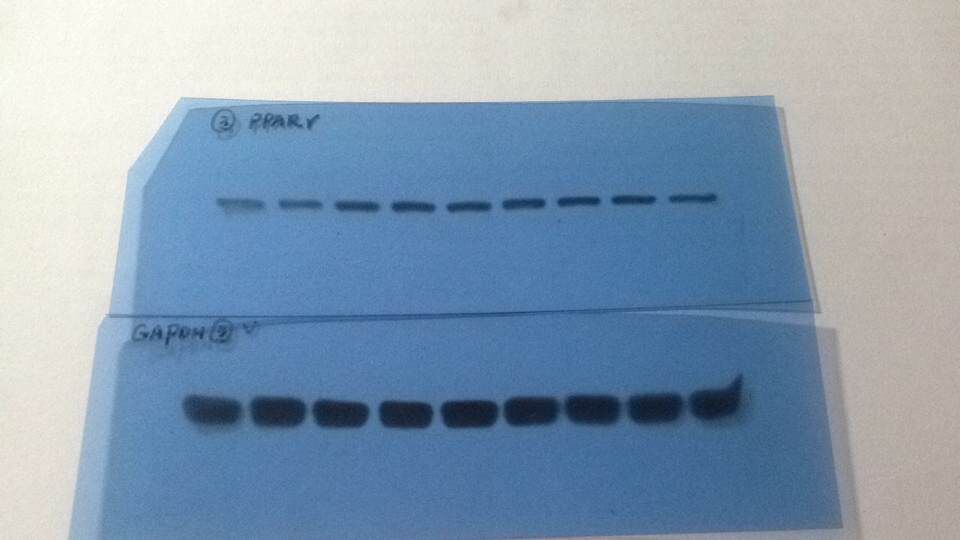


4.


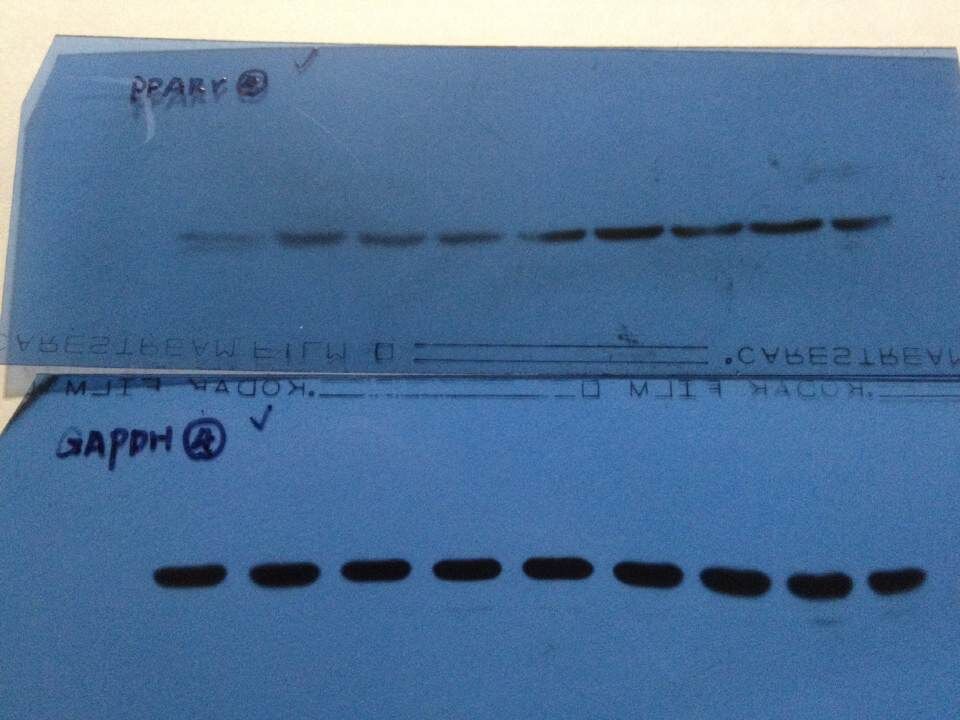


5.


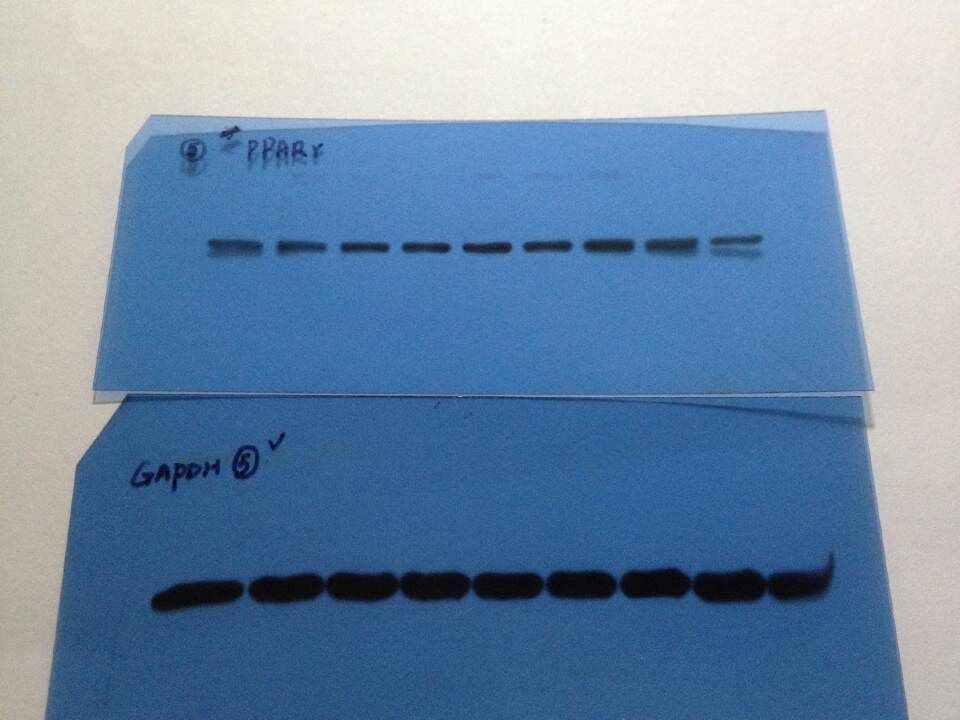


6.


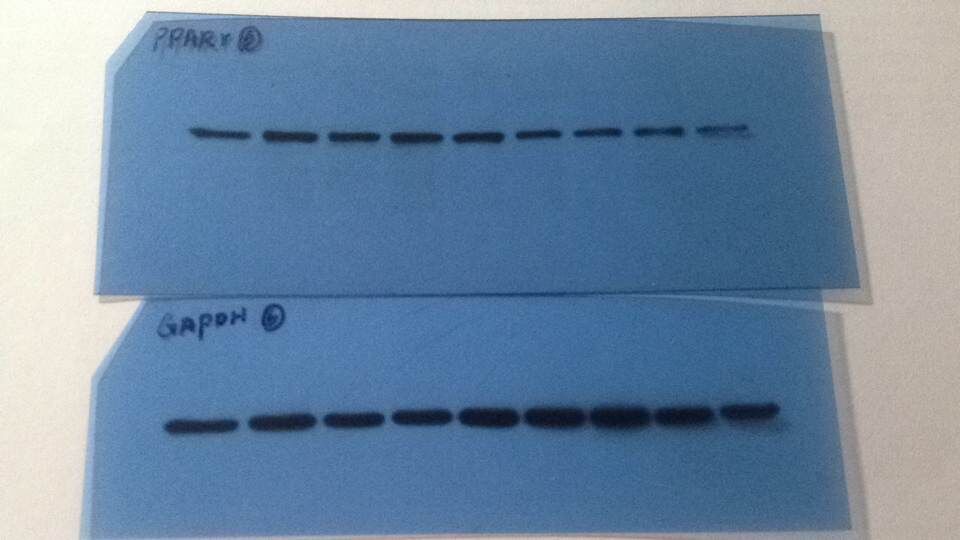


7.


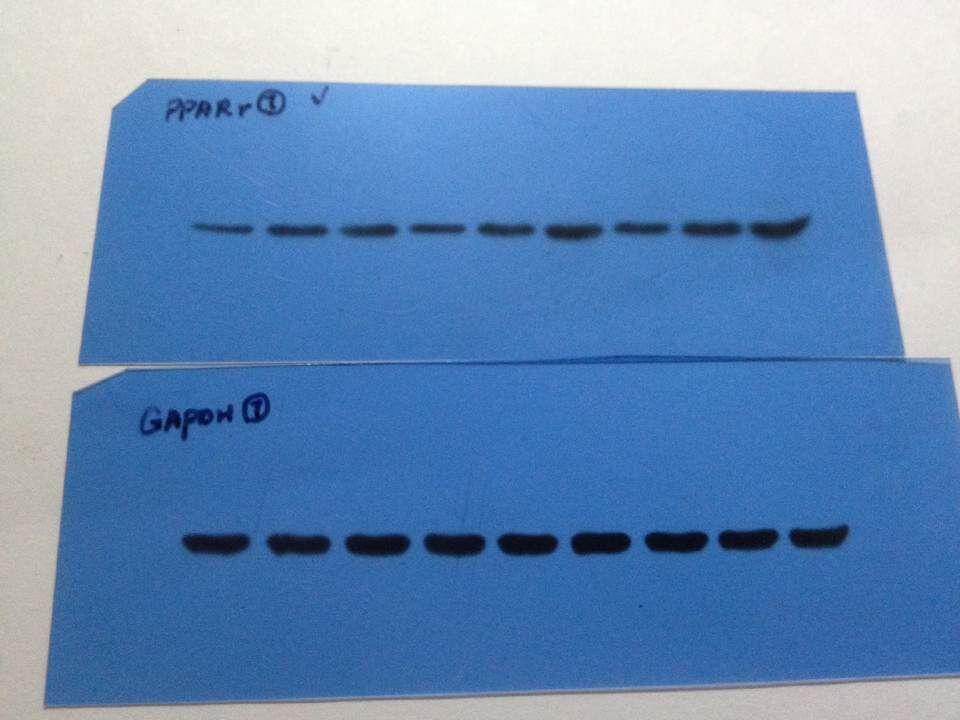


8.


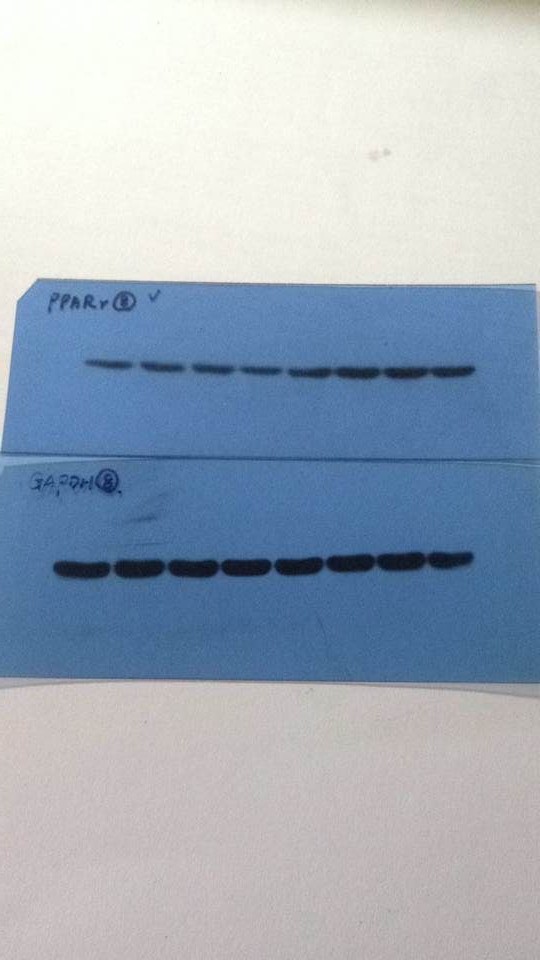


The sequence of western blot showed here.

1. 1-2-3-4-5-6-7-8-9
2. 10-11-12-13-14-15-16-17-18
3. 19-20-21-22-23-24-25-26-27
4. 28-29-30-31-32-33-34-35-36
5. 50-51-52-53-54-55-56-57-58
6. 37-38-39-40
7. 41-42-43-44-45-46-47-48-49
8. 59-60

Relationship between the sequence and experimental groups is as follows.

1h reperfusion subgroup: FNS group: 40, 49, 84, 60, 38

Control group: 39, 48, 85, 59, 37

3h reperfusion subgroup: FNS group: 10, 7, 35, 50, 42

Control group: 9, 1, 36, 51, 41

6h reperfusion subgroup: FNS group: 5, 12, 19, 33, 3

Control group: 6, 11, 20, 34, 4

9h reperfusion subgroup: FNS group: 16, 17, 14, 8, 56

Control group: 15, 18, 13, 2, 57

12h reperfusion subgroup: FNS group: 22, 23, 26, 45, 58

Control group: 21, 24, 27, 44, 43

15h reperfusion subgroup: FNS group: 29, 31, 55, 53, 25

Control group: 28, 30, 54, 52, 32
